# Efficacy of repeated intrathecal triamcinolone acetonide application in progressive multiple sclerosis patients with spinal symptoms

**DOI:** 10.1186/1471-2377-4-18

**Published:** 2004-11-07

**Authors:** Kerstin Hellwig, Franz Josef Stein, Horst Przuntek, Thomas Müller

**Affiliations:** 1 Department of Neurology, St. Josef Hospital, Ruhr University Bochum, Gudrunstrasse 56, 44791 Bochum, Germany

## Abstract

**Background:**

There are controversial results on the efficacy of the abandoned, intrathecal predominant methylprednisolone application in multiple sclerosis (MS) in contrast to the proven effectiveness in intractable postherpetic neuralgia.

**Methods:**

We performed an analysis of the efficacy of the application of 40 mg of the sustained release steroid triamcinolone acetonide (TCA). We intrathecally injected in sterile saline dissolved TCA six times within three weeks on a regular basis every third day in 161 hospitalized primary and predominant secondary progressive MS patients with spinal symptoms. The MS patients did not experience an acute onset of exacerbation or recent distinct increased progression of symptoms. We simultaneously scored the MS patients with the EDSS and the Barthel index, estimated the walking distance and measured somatosensory evoked potentials. Additionally the MS patients received a standardized rehabilitation treatment.

**Results:**

EDSS score and Barthel index improved, walking distance increased, latencies of somatosensory evoked potentials of the median and tibial nerves shortened in all MS patients with serial evaluation (p < 0.0001 for all variables). Side effects were rare, five patients stopped TCA application due to onset of a post lumbar puncture syndrome.

**Conclusions:**

Repeated intrathecal TCA application improves spinal symptoms, walking distance and SSEP latencies in progressive MS patients in this uncontrolled study. Future trials should evaluate the long-term benefit of this invasive treatment.

## Background

There are controversial results on the efficacy of the nowadays still abandoned, intrathecal steroid application predominantly due to a missing detailed evaluation of patients' characteristics, careful monitoring and standardized outcome measurements in multiple sclerosis (MS) [1]. Initially, case reports of intrathecal methylprednisolon and ACTH administration described beneficial effects in MS patients, but the following studies showed disappointing results in particular in comparison to systemic steroid application [1]. Earlier intrathecal treatment trials in MS patients suffered from small sample sizes, low number of injections, low steroid dosages, short half life of the administered steroid and an increasing number of reports on side-effects, i.e. adhesive arachnoiditis and various forms of meninigitis probably due to neurotoxic solvents and bacteriostatic additives [4]. Moreover a retarded release steroid preparation was not available for many years [1,5]. Then administration of triamcinolone acetonide crystal suspensions (TCA), dissolved at bedside in sterile saline, was introduced in the intrathecal steroid treatment of MS. However, studies again lacked of detailed evaluation and clinical characterization of MS patients, small sample sizes and a low number of intrathecal application of this retarded release steroid compound [1,4,5].

Accordingly, there was no convincing superiority over the efficacy of the systemic steroid treatment. Some enrolled MS patients experienced a recent deterioration of symptoms due to prior acute relapses and/or ongoing chronic progression. Moreover study participants were not classified according to the various subtypes of MS progression [5,6]. However in general, a comparison of both methods of steroid application is at least doubtful from the pharmacokinetic point of view. The resulting steroid efficacy in the central nervous system enormously differs in favor for the intraspinal application due to the achieved cerebrospinal fluid steroid level, marked longer half life, i.e. with detection of TCA even four months after the last administration, and missing impact on the endogenous peripheral cortisol secretion with no appearance of side effects of systemic high dosage steroid application [4,6]. In recent years, a certain revival of intrathecal methylprednisolon administration took place in the treatment of intractable postherpetic neuralgia and MS with spinal symptoms, both of which turned out to be very effective, but were controversially discussed regarding the safety issues [2,3]. The MS study demonstrated, that six repeat intrathecal TCA injections within three weeks reduced the EDSS score in 31 of 36 progressive MS patients with predominant spinal symptoms. 20 of them entered a follow-up period of 13.1 ± 6.22, 3 – 23 [mean ± SD, range] months with 6.35 ± 3.91, 2 – 15 TCA injections. They received one TCA application on a regular basis in an individually differing frequency every six to twelve weeks. These patients remained stable [3]. Nevertheless, there is a need for further results on the usefulness of this treatment. The optimum design would be a placebo-controlled arm, but repeat performance of intrathecal saline (placebo) administration under double-blind conditions with the patients' consent and an approval of an ethical committee is not realistic in clinical practice. Moreover they may be ethical concerns of withholding treatment [7]. Our present study is a way out of this dilemma of the debate on the efficacy of TCA treatment, which is carried out in certain specific centers in Germany for many years now [3]. We performed an analysis of standardized intrathecal application of TCA in MS patients with spinal symptoms, using subjective rating procedures and objective measurements.

## Methods

### Subjects

We only enrolled clinically well characterized, consecutively referred MS patients (table 1, 2) with distinct spinal symptoms and/or MRT visualized lesions in the spinal cord [8]. These patients did not suffer from an acute onset of exacerbation or recent clearly increased progression of their symptoms.

**Table 1:** Patients' characteristics.

Age	50.10 ± 10.30, 21 – 78 years
Duration of MS	14.32 ± 7.63, 2 – 40 years
Sex	119 men, 42 women
MS types	chronic progressive: n = 35
	secondary progressive: n = 122
	relapsing-remitting: n = 4
Length of hospital stay	28.41 ± 5.97; 21 – 60 days

**Table 2:** Treatment against spasticity.

	Before TCA	After TCA
Baclofen	7.56 ± 16.06; 0 – 80 mg, n = 117 without baclofen	6.44 ± 15.51; 0 – 80 mg, n = 125 without baclofen
Tolperison	1.19 ± 8.20; 0 – 75 mg, n = 154 without tolperison	1.34 ± 7.41; 0 – 50 mg, n = 152 without tolperison
Tizanidin	1.94 ± 5.29; 0 – 32 mg, n = 132 without tizanidin	3.38 ± 6.64; 0 – 32 mg, n = 110 without tizanidin

All data are given as mean ± standard deviation; minimum – maximum; n = number of patients; TCA = standardized intrathecal triamcinolon acetonide application according to the methods section.

### Methods

We performed scoring with both, EDSS and Barthel index and assessed the walking distance. Then we measured somatosensory evoked potentials (SSEP) in a standardized fashion before start and at the end of the intraspinal TCA treatment within a prospective study design [9]. A technician performed SSEP recordings and measured the walking distance. We blinded the EDSS raters. Retrospectively, we compiled information on patients from their hospital records, i.e. date of birth, sex, duration of disease after diagnosis of MS, dosages of oral baclofen (lioresal^®^), tolperison (mydocalm^®^), tizanidin (sirdalud^®^) on the first and last day of the hospital stay, length of hospitalization in days (tables 1 &2). The patients additionally received a standardized rehabilitation treatment, which included physiotherapy, massage and optional swimming with the patients' consent [12,13]. Only data with successfully performed serial evaluation of patients were compared for each variable. We performed lumbar puncture with an "atraumatic" Sprotte needle [10,11]. Each patient received six intrathecal applications of 40 mg TCA followed by a mandatory stay in bed for at least six hours. This should reduce incidence of lumbar puncture syndrome and hypothetically support the diffusion of TCA in the CSF and the spinal cord [14,15]. A preexisting immune system modulating drug therapy remained stable. We closely monitored for typical concomitant with systemic steroid application appearing side effects, i.e. increase of body weight etc., all of which did not significantly change (data not shown) [16]. No slight or severe side effects occurred, but we did not include five patients into our evaluation due to onset of post lumbar puncture syndrome with headache and nausea, which caused a stop of further intrathecal TCA applications. These patients withdraw their consent. We only considered SSEP data of patients with serial measurements, which we performed on the same day of the EDSS rating.

### Ethics

Each participant gave written informed consent for the TCA treatment, which was approved by the local ethical committee. The consent form included a detailed description of all putative risks of lumbar puncture and intrathecal TCA application.

### Statistics

Data showed a normal distribution according to the Kolmogorow-Smirnow test. As a result, we only performed parametric tests. We used ANCOVA with repeated measures design including MS duration, MS types, change of dosages of concomitant drugs against spasticity, length of hospital stay, sex and age as covariates. We computed SSEP results by adding both sides in order to reduce amount of calculations for comparisons. Then we calculated the differences of latencies between both timepoints of recordings according to the formula [Initial - End = Diff] for correlation analysis. We employed linear regression for correlation analysis. Level of significance of p-values were adjusted to 0.05 divided by the number of performed comparisons respectively correlations.

## Results

### Comparisons

EDSS score (n [number of subjects with serial evaluation] = 161) and Barthel index (n = 68) improved and walking distance (n = 161) increased (table 3). SSEP latencies of tibial (n = 136) and median (n = 108) nerves reduced (table 3). P-values of all performed comparisons were below 0.0001. No significant effects of covariates appeared.

**Table 3:** Comparison of clinical data.

	Before TCA	After TCA	F
EDSS – score	6.44 ± 1.06; 3.5 – 6.5	5.47 ± 1.24; 2 – 8.5	379.28
walking distance	158.03 ± 501.20; 0 – 5000	439.38 ± 895.24; 0 – 5000	34.40
Barthel index	58 ± 20.07; 5 – 100	89.13 ± 12.57; 60 – 100	347.52
P2 (tibial nerve)	105.80 ± 10.38; 85 – 130	88.57 ± 5.60; 77 – 106	470.96
N2 (tibial nerve)	118.83 ± 10.49; 92 – 148	101.63 ± 6.43; 80 – 118	425.71
P3 (tibial nerve)	131.79 ± 13.22; 70 – 164	114.31 ± 7.46; 95 – 135	325.88
N2 (median nerve)	46.53 ± 5.40; 23–60	41.03 ± 2.92; 23–47	138.10
P2 (median nerve)	52.73 ± 6.34; 25 – 68	47.09 ± 3.03; 41–56	128.80

All data are given as mean ± standard deviation; minimum – maximum in the second and third column; latencies (N2, P2, P3) of the somatosensensory evoked potentials are given in milliseconds, walking distance is given in meters, F = F-value of ANCOVA, SD = standard deviation; TCA = standardized intrathecal triamcinolon acetonide application according to the methods section.

### Correlation analysis

There were no significant relations between computed changes of EDSS scores, walking distances and SSEP results (results not shown). Diff P2 correlated with Diff P3 (R [correlation coefficient] = 0.71), Diff P2 with Diff N2 (R = 0.90), Diff P3 with Diff N2 (R = 0.74) of SSEP latencies of the tibial nerves. There were relations between Diff N2 and Diff P2 (R = 0.85) of SSEP latencies of the median nerves. P-values of these correlations were below 0.0001. Moreover Diff P2 of the tibial nerves correlated with Diff N2 of the median nerves (R = 0.28, p = 0.004, n = 100). There was a certain trend for a significant correlation between Diff P2 of the tibial nerve and Diff P2 of the median nerve (R = 0.22, p = 0.03). No other significant associations of SSEP data appeared (results not shown).

## Discussion

Our results demonstrate and confirm the efficacy of repeated intraspinal TCA application in MS patients with spinal symptoms, which improved according to the EDSS outcomes and the results of assessed walking distance and determined SSEP latencies [3]. We intrathecally injected TCA six times within three weeks, whereas earlier trials weekly performed one application up to three times at the most [4]. The distinct reduction of SSEP peak latencies and the significant relations between their computed differences confirm the clinical outcomes and underline the efficacy of intraspinal TCA treatment in MS patients with spinal symptoms in general. We hypothesize, that these neurophysiological results indicate a certain remyelinating and/or restorative potential of intraspinal TCA application with an at least transient shift from chronic inflammation to remyelination [17,18]. Our results support the crucially discussed view, that serial SSEP studies in MS may monitor the effect of treatment to a certain extent under standardized conditions [9,19,20]. Our analysis also shows that primary and secondary progressive even advanced MS patients with spinal symptoms predominantly improve from this kind of intrathecal steroid therapy. We assume, that we achieve persistent high steroid concentrations at lesions of the spinal cord, since TCA must not pass the blood brain barrier [6]. However previous comparisons of the clinical efficacy of intrathecal TCA application with the intravenous administration of methylprednisolone showed no superiority of one method over the other [5,6,19]. But these studies did not exclude relapsing remitting patients or participants with a previous acute relapse. They did not focus on spinal symptoms. Their application rate of TCA was distinct lower compared with the one of our present and a previous trial [3].

However our present study outcomes do not allow any conclusions on the duration of the achieved benefit and the impact of TCA treatment on progression of MS [3]. Therefore there is an urgent need for further confirmatory trials, which additionally address all these issues. A strategy would be to choose one arm with active treatment and one arm with just follow-up without active treatment with blind assessment by an evaluating physician. However we stress concerning long-term steroid therapy and progression of MS, that there are positive outcomes of trials with intravenous methylprednisolone administration in various application rates and dosages on long term disease progression and/or on brain atrophy in secondary-progressive -, respectively relapsing-remitting MS patients [16,21]. In contrast to studies on intravenous oral steroid treatment, we did not observe the typical side effects of systemic high dosage steroid administration, i.e. edema. This may support previous findings by circumstantial evidence, which report no decrease of endogenous cortisol secretion following intrathecal TCA administration [4].

We cannot exclude a certain impact of physiotherapy, the standardized rehabilitation treatment and an beneficial effect of hospitalization in general with its resulting concomitant positive influence on activities of daily living [12,13]. However, we found no significant impact of the corresponding covariate length of the hospital stay in our statistical analysis. We assume, that our results do not reflect an improved drug therapy against spasticity, since no significant impact of the covariate computed changes of medication appeared. Nevertheless we cannot exclude a certain effect of the steroid on spasticity. However most participants did not take any drug against spasticity. Onset of side effects of lumbar puncture itself were negligible, since we used an atraumatic needle [11].

## Conclusions

Our data demonstrate the efficacy and safety of repeated intrathecal TCA application in MS patients with predominant spinal symptoms, which markedly improved. Some MS patients experienced post lumbar puncture syndrome with a frequency within the normal range [11], but typical side effects of systemic high dosage steroid administration did not appear.

## Competing interests

The author(s) declare that they have no competing interests.

## Authors' contributions

KH, FS, HP and TM designed, coordinated and carried out the study. TM performed statistical data analysis and drafted the manuscript. All authors read and approved the final manuscript.

## Pre-publication history

The pre-publication history for this paper can be accessed here:


